# Development of RT-qPCR for quantification of human enterovirus D68 *in vitro*

**DOI:** 10.1016/j.mex.2023.102234

**Published:** 2023-05-30

**Authors:** Hanne Lillerovde Ørstenvik, Ann-Kristin Tveten, Yanran Cao

**Affiliations:** Faculty of Natural Sciences, Department of Biological Sciences Ålesund, Norwegian University of Science and Technology, Norway

**Keywords:** *Reverse transcription-quantitative polymerase chain reaction (RT-qPCR) assay*, Human Rhinovirus 87, Enterovirus D68, Respiratory infection, Real-time PCR, Quantification, Detection

## Abstract

The common cold is the most frequent viral infectious disease of the upper respiratory tract with different intensities based on the serotype and the characteristics of the virus. Numerous human rhinoviruses have been identified and classified. Human rhinovirus 87 (HRV87), also known as enterovirus D68 (EV-D68), is one of the common viruses causing respiratory infections. In this study, a reverse transcription-quantitative polymerase chain reaction (RT-qPCR) assay was developed, optimized, and validated for the detection of EV-D68. Method development also covers specificity, sensitivity, efficiency, and inter-and-intra-assay variations. Overall, this one-step qPCR assay will permit quantitative assessments of human enterovirus D68 RNA.

• Enterovirus D68 is a reemerging viral agent causing respiratory infection.
• RT-qPCR assay developed for detection of human enterovirus D68.
• In this article validation to secure reproducibility is done according to MIQE guidelines.

## Highlights

• One-step RT-qPCR assay for fast and specific detection of human enterovirus D68.
• Primer sets designed specific for human enterovirus D68.
• Assay validation according to MIQE guidelines.

## Graphical abstract

**Figure:**
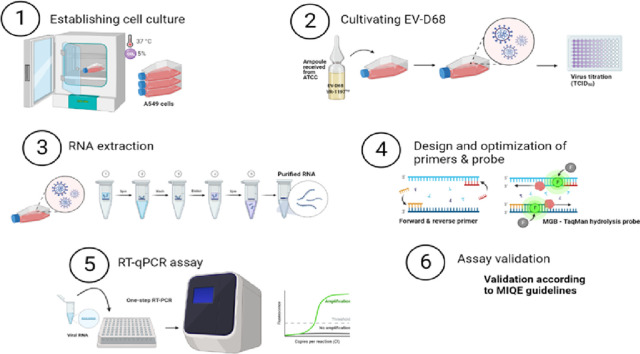

Specifications Table

**Table:** 

Subject area:	Immunology and Microbiology
More specific subject area:	*Viral detection*
Name of your method:	*Reverse transcription-quantitative polymerase chain reaction (RT-qPCR) assay*
Name and reference of original method:	*Not Applicable (N/A)*
Resource availability:	*Reagents and Equipment are listed in the Materials section*

## Background

Human rhinovirus is a common virus causing respiratory illnesses mostly in the upper respiratory tract. It is also known as the agent causing the majority of respiratory infections referred to as the common cold [1], [2], [3]. Throughout the years over a hundred different rhinovirus serotypes have been identified and described. The different serotypes have been sorted according to the surface receptor necessary for attachment and entry into the cell. Receptors used by rhinoviruses are divided into one of two families: the major receptor and minor receptors [4,5].

Human Rhinovirus 87 (HRV87) contradicts the description of other rhinovirus serotypes because it has not been reported to use either of the two receptor families typical for rhinoviruses. Instead, HRV87 use sialic acid found in glycolipids and glycoproteins in the membrane surrounding cells as the primary entry receptor [6,7]. Also, the characteristics and genetic possessions of HRV87 have properties typical of both enteroviruses and rhinoviruses [1,5]). Human rhinovirus 87 is more resistant to an acidic environment and lower temperatures compared to other rhinovirus strains [8], [9], [10]. These characteristics are similar to another virus in the picornaviridae family, the human enterovirus D68 (HEV-D68) [1,8]. It was realized that these characteristics refer to that there is high degree of nucleotide identity (95%) between HRV87 and EV-D68, indicating that they are very closely related. Based on this, they are now considered the same virus. The genetic structure falls somewhere between HRVs and EVs indicating the need for developing a specific PCR detection method [1,[8], [9], [10], [11]).

Enterovirus D68 is a virus causing infections in the respiratory tract. The clinical feature describing EV-D68 ranges from severe cases that need medical attention, to cases without any symptoms. EV-D68 most commonly cause symptoms we know as the common cold. Typically including fever, sore throat, sneezing and muscle pain [12]. However, the virus also causes infection in the gastrointestinal tract and can result in neurological complications [11,10].

EV-D68 is considered a risk factor when it comes to public health, especially after the outbreak in 2014. The virus cause infections in the respiratory system that can spread fast from one host to the other. It has also been reported that EV-D68 is connected to acute flaccid myelitis (AFM) which has a neurological effect that weakens muscles. Because of this, it is of high importance to better understand the virus itself [12].

This study aims to develop and validate an RT-qPCR assay for the quantification of EV-D68. Validation is done according to the Minimum Information for Publication of Quantitative Real-Time PCR Experiments (MIQE) guidelines, determining the efficiency, sensitivity, specificity and inter-and-intra-assay and variability [13].

## Method details

### Cultivate cells

Human lung (A549) cells were purchased from American Type Culture Collection (ATCC, CCL-185). The cells were kept at – 80 °C before cultivation.

1. In a laminar flow cabinet make growth media according to the instructions described for the cell line [14]. Growth media consist of DMEM/F-12 medium (Gibco, Thermofisher, US) supplemented with 10% Fetal Bovine Serum (Gibco, Thermosteric, US) and 1% Antimycotic Antibiotic 100X (Gibco, Thermofisher, US).
2. Adjust pH to fit the described levels for the optimal growth conditions (pH 7.42).
3. Set the incubator to the appropriate temperature and CO_2_ level according to optimal growth conditions for the cell line.
4. A549 human lung carcinoma epithelial cells were cultivated in adherent culture at 37 °C and in a humidified atmosphere containing 5% CO_2_.
5. Control cell growth and make sure the conditions are optimized.
6. Maintain cell cultivation to ensure the correct development and for further virus cultivation. Split cells when confluent > 80%.

### Cultivate virus

Human Enterovirus D68 (EV-D68 (HRV87)) (VR-1197™) with taxonomy ID: 167,331 were purchased from the American Type Culture Collection (ATCC, Manassas, VA). The virus was obtained at −80 °C before cultivation.

The procedure for virus cultivation according to the ATCC virology was used [15].

1. Work in a biosafety cabinet. Prepare a cell flask with a confluent level > 70%. Remove the old growth medium before adding PBS to wash the cell monolayer.
2. Manage viral ampule according to the specific description in agreement to hazard level.2.
3. Thaw the viral ampule in a water bath at 37 °C and decontaminate the ampule.
4. Virus (1 ml) is added to 19 ml medium in a 50 ml centrifuge tube. 1 ml from the centrifuge tube is added to a cell flask with 9 ml growth medium.
5. The culture flask is placed in the incubator at 33 °C. The CPE appeared as round and refractile cells distributed in small clades in different layers in the cell culture could be observed four days past infection (DPI).

### Viral titration

Median Tissue Culture Infectious dose (TCID_50_) is conducted according to the ATCC procedure for virus propagation in the ATCC virology procedure [17]. Work in a biosafety cabinet in required. A ten-fold dilution with 10° – 10^−8^ range is made. Virus titer assay, TCID_50_, is carried out to determine a value for which EV-D68 concentration leads to infection in 50% of A549 cells. The TCID_50_ value is calculated based on the number of lysed cells at various concentrations.

1. **Seed culture plate with host cells**
  Seed 5000 cells per well in 200 µl growth media on each well, rows 2–11 of 96-well tissue culture plate (VWR, no). Add 200 µl growth medium without cells in row 12. Gently rock plates back and forth and from side to side so that cells are distributed evenly. Once cells have been seeded, allow the cells to grow overnight. The next day, cells are visualized under a light microscope to confirm that cells are evenly distributed and reached over 80% confluency.
2. **Prepare serial dilutions of viruses**
  Make a series of dilutions at 1:10 of the original virus sample. Fill all the tubes in series with 3.6 ml growth medium. Briefly mix the original virus stock and transfer 400 µl of the virus to the first tube. Then transfer 400 µl of the diluted virus to the second tubes in the series. Repeat to make a serial 1:10 dilution of the virus, e.g., such as 10^−3^ through 10^−7^.
3. **Infect monolayer cells**
  Label the lid of the 96-well dish by drawing grid lines to define quadruplicates and number each grid to correspond to the virus sample and label the rows of the plate for the dilution that will be plated. Row 1 is negative wells that will not be infected. Carefully remove all media from each well. Add 200 µl of virus dilution per well, proceeding backwards through the dilutions. Allow the virus to adsorb to cells by placing the plate back in the incubator at 33 °C for monitoring CPE for 5 days.
4. **Visualization and calculation of TCID_50_**
  The endpoint is determined when the CPE read-out appears the same per dilution for 3 separate readings. Medium is removed, and cells are washed with 100 µl of Dulbecco's phosphate buffered saline (DPBS, Sigma, US) and fixed by adding 50 µl of methanol per well for 1 min. The methanol is then replaced with 100 µl of 0.1% crystal violet in distilled water for 20 min. Plates are washed, and absorbance at 570 nM is measured using a plate reader. The amount of specimen required to infect 50% of monolayers (TCID50) is determined. The titer is calculated using the method of Muench and Reed [16].

### RNA extraction

The viral RNA was extracted directly according to the RNeasy Plus Mini Kit (Qiagen, Valencia, CA) procedure [17]. EV-D68 was isolated from a cell flask with virus cultivated for 7 days with 100% CPE. Prior to sample isolation, 350 µl RLT buffer is added to a 2 ml eppendorf tube. The use of a G-column was excluded from the procedure.

1. From the viral sample, 100 µl is added to the RLT buffer.
2. Add 1 volume (450 µl) of 70% ethanol added to the Eppendorf tube. Vortex mix Eppendorf tube gently.
3. Transfer up to 700 µl of the sample to the Rneasy Mini spin column placed in a 2 ml collection tube. Close the tube and centrifuge for 15 s at ≥ 8000 x g. Flow-through is discarded.
4. Repeat the previous step for the remaining sample. Place the Rneasy spin column into a new collection tube.
5. Add 700 µl RWI buffer to the RNeasy spin column. Close the lid, and centrifuge for 15 s at ≥8000 x g. Discard the flow-through.
6. Add 500 µl RPE buffer to the RNeasy spin column. Close the lid, and centrifuge for 15 s at ≥8000 x g. Discard the flow-through.
7. Add 500 µl RPE buffer to the RNeasy spin column. Close the lid, and centrifuge for 2 min at ≥8000 x g.
8. Transfer the Rneasy spin column to a new 1.5 ml. Add 40 µl RNase-free water close the lid, and centrifuge for 1 min at ≥8000 x g. The RNeasy spin column is discarded, and the tube is closed.
9. The RNA sample is placed directly at – 80 °C. To control the quality of RNA extraction, Qubit 4.0 Fluorometer (Invitrogen, Thermofisher Scientific, US) is used to measure the concentration (not shown) [18].

### Primer & probe design

**Fig. 1:**
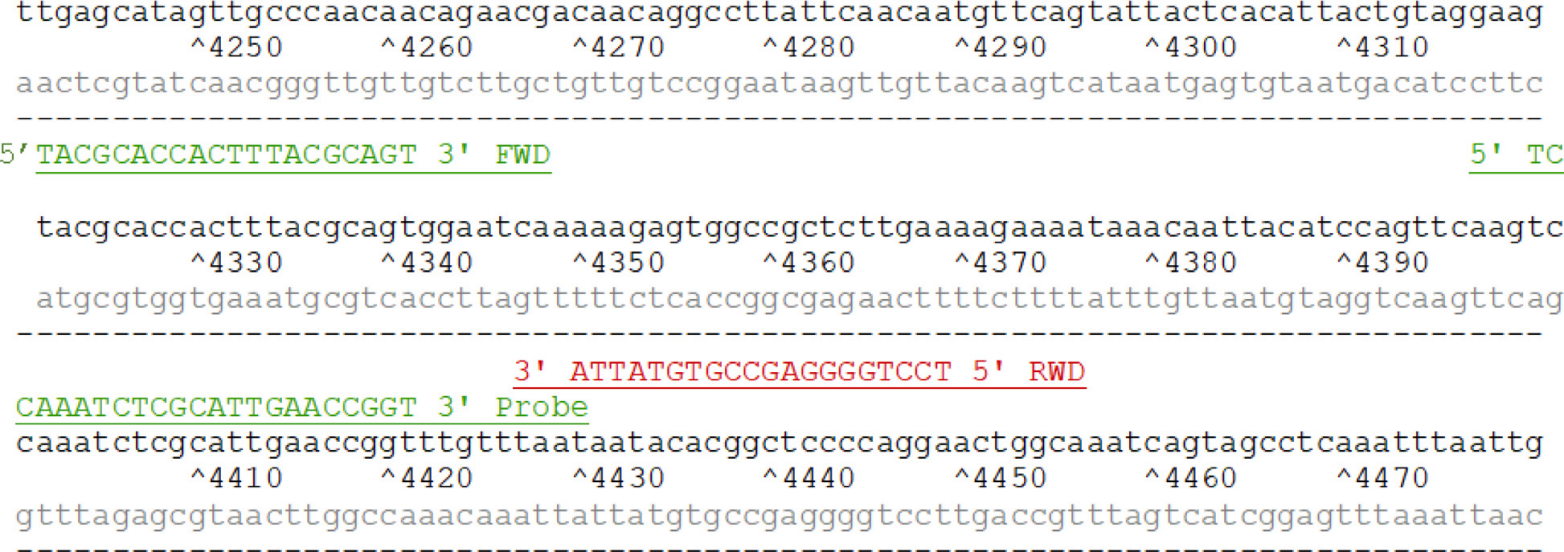
shows the nucleotide sequences for the forward primer (FWD), reverse primer (REV) and the probe for the human enterovirus D68 detection.

Forward primer (FWD) and reverse primer (RWD) were designed through the publicly available primer design in National centre for Biotechnology Information (NCBI) [19]. Primers are designed to amplify (the target regions of) the partial polyprotein (139 aa) in the human enterovirus D68 (NCBI: txid167331). The nucleotide sequences designed are presented in Fig. 1. Full lineage in NCBI: Riboviria; Orthornavirae; Pisuviricota; Pisoniviricetes; Picornavirales; Picornaviridae; Ensavirinae; Enterovirus; Enterovirus D; enterovirus D68.

### Primer & probe optimization

The primers and probe are optimized within a 200 nm - 600 nm range. Optimization was conducted to determine the concentrations necessary for EV-D68 quantification. The qScript XLT one-step RT-qPCR though mix®, low ROX™ (Quantabio, Beverly, US) was used in the optimization. Primer and probe sequence, modifications and merchant are listed in Table. 1.

1. Dilute forward primer (FWD) & reverse primer (RWD) to 100 nM concentration.
2. Experimental review of primer concentrations in the range 200nM-600 nM. Probe concentration set to 200 nM while optimizing primers.
3. Optimization of the probe takes place after primer optimization in the range of 100nM-600 nM.
4. Evaluate the best primer combination based on PCR slope and C_q_ value.

**Table 1:** show the primers and probe designed for the amplification of human enterovirus D68. 5`−3`sequence of the Minor Groove Binding (MGB) probe from Applied Biosystems and its modifications are presented. The sequence, modification, and merchant of the reverse primer (RWD), forward primer (FWD) and probe are also listed.

Type	Sequence 5`- 3`	Modifications	Merchant
MGB probe	TCC AAA TCT CGC ATT GAA CCG GT	6-FAM MGBNFQ	Applied Biosystems UK (United Kingdom)
RWD	TCC TGG GGA GCC GTG TAT TA	–	Invitrogen
FWD	TAC GCA CCA CTT TAC GCA GT	–	Invitrogen

### Reverse transcription - qPCR

For the RT-qPCR, the qScript XLT one-step RT-qPCR though mix®, low ROX™ (Quantabio, Beverly, US) master mix was chosen. The choice of master mix makes the PCR a collective one-step process. The reaction volume for each PCR run is set to 15 µl based on in-house experience with PCR. Here, the methodology is a specific probe-based detection using the hydrolyzed MGB probe. Table 2

1. The first step in establishing a methodology for detection is to set up a run to assess the functionality of the reagents. Optimize reagent volumes and concentrations for each well as shown in Table. 3.

**Table 3:** shows the six components necessary to conduct RT-qPCR for human enterovirus quantification. The final concentration and volume of the reagent; master mix, probe, reverse primer (RWD), forward primer (FWD), genetic material (RNA) and distilled water (ddH_2_O) are presented. Altogether the total volume for each reaction is 15 µl.

Reagents	Concentration	Amount (µl)
Master mix	1X	7.5
ddH_2_O	1X	2.6
Probe	300 nM	1.8
RWD primer	400 nM	1.2
RNA	1X	1.0
FWD primer	300 nM	0.9
2. Include positive and negative controls on each PCR plate.
  a. Reference dye ROX as an internal control.
  b. No template control (NTC) is the negative control to assess the reagents.
  c. A positive viral sample works as a positive control to evaluate the assay.
3. Plan how to prepare the PCR plate. Set up samples with biological and technical replicates.
4. Prepare a 10-fold dilution for the standard curve and limit of detection (LOD).
5. Pipette reagents in 96-MicroAmp plates (0.1 ml Applied Biosystems) and seal it with adherent film (Microamp Optical Adhesive Film, Applied Biosystems) to secure the reaction volume.
6. Place plates in a plate spin II centrifuge from ThermoFisher Scientific for 40 s at 34 × 100 rmp.
7. The PCR plate is inserted into AriaMx Real-Time PCR (qPCR) instrument from Agilent Technologies with connecting software [20].
8. Set qPCR amplification cycle as seen in Fig. 2.

**Fig. 2:**
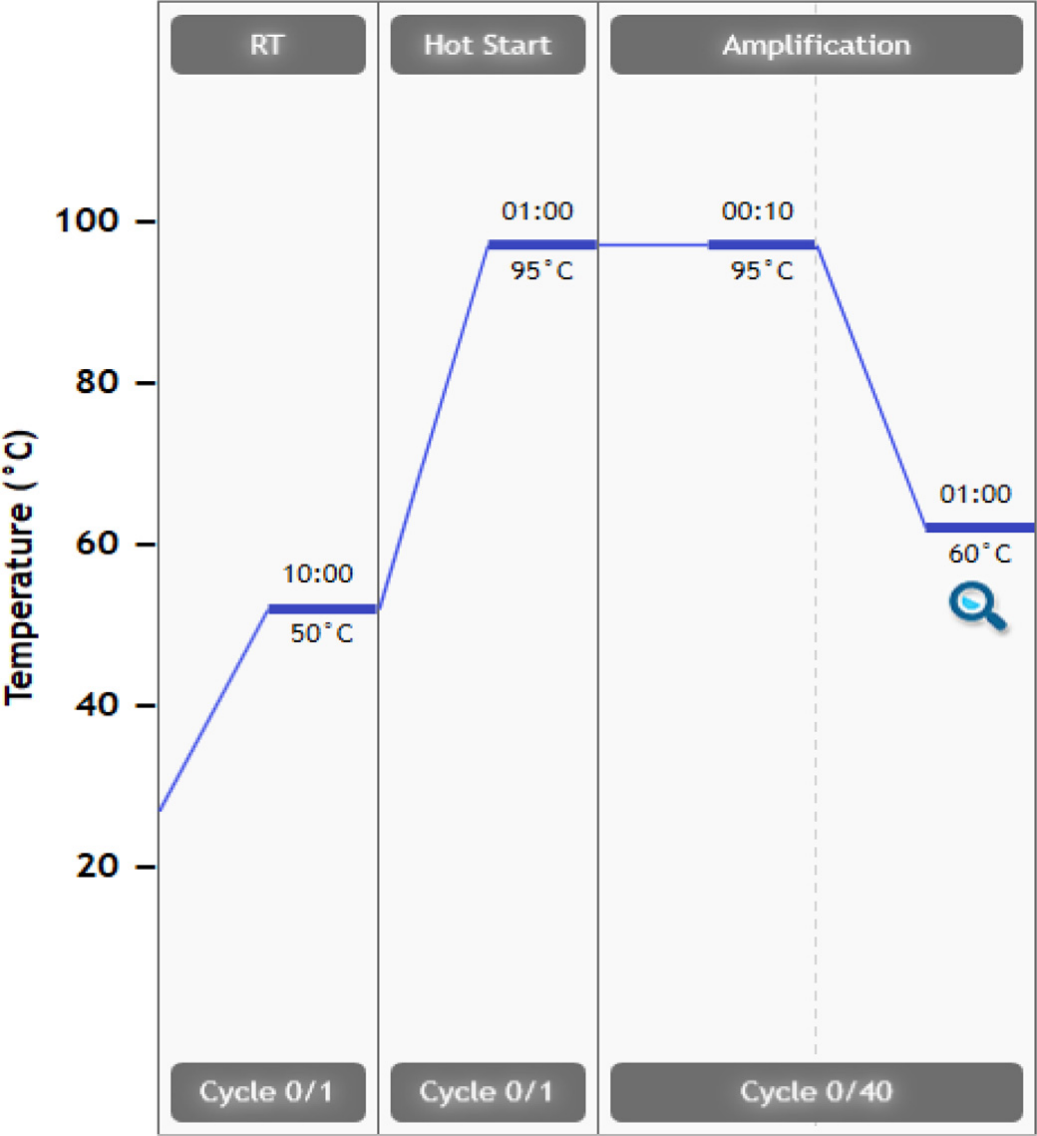
shows the different temperature-regulated steps during the RT-qPCR. The amplification cycle consists of a total of 40 cycles. The first step is the Revers Transcription step where RNA is converted to DNA at 50°. Followed by a Hot start for the amplification of the genetic material.
  The qPCR amplification cycle was developed with the RT-step where cDNA synthesis was conducted for 10 min at 50 °C. After the initial denaturation step for 1 min at 95 °C, the PCR reaction was carried out under the following conditions: 95 °C for 10 s, 60 °C for 1 min for 40 cycles.
9. Evaluate slope and register C_q_ values for each well.

**Table 2:** Show an overview of how to assess the different primer concentrations.

Forward /Reverse Primer	200 nM	300 nM	400 nM	500 nM	600 nM
200 nM					
300 nM					
400 nM					
500 nM					
600 nM					

## Assay validation

The MIQE guidelines [13] refer to a common standard that is used in work to establish new PCR methods. For the preparation of RT-qPCR, the method must be validated based on sensitivity, specificity, precision, and efficiency. The validation parameters are evaluated based on the choice of reagents, optimization of primer and probe, and establishment of a standard curve. In this study, we first validated the assay in a one-step RT-qPCR format using serial dilutions of an *In Vitro* transcribed RNA reference. One-step RT-qPCR was decided to use throughout the study because the advantages of limited contamination and simplicity account for better conditions than the disadvantages compared to two-step RT-qPCR. For one-step reactions, all reagents are added in the same well on the PCR plate. Conversion from RNA to the more stable cDNA takes place internally in a closed environment and as a result, excludes consideration of the reverse transcriptase step and controls that convert RNA into cDNA.

### Efficiency

The standard curves R-value and slope provide information on the robustness and effectiveness of the method. To assess the effectiveness of RT-qPCR, the amplification of the target gene was assessed. For each cycle (n) a backup takes place. It is desirable that for each cycle the amplification should produce 2n copies. Ideal copying, meaning 100% efficiency in RT-qPCR gives a slope between −3.0 and −3.8. At the same time, it is crucial that R^2^ > 0.99 for efficiency.

**Fig. 3:**
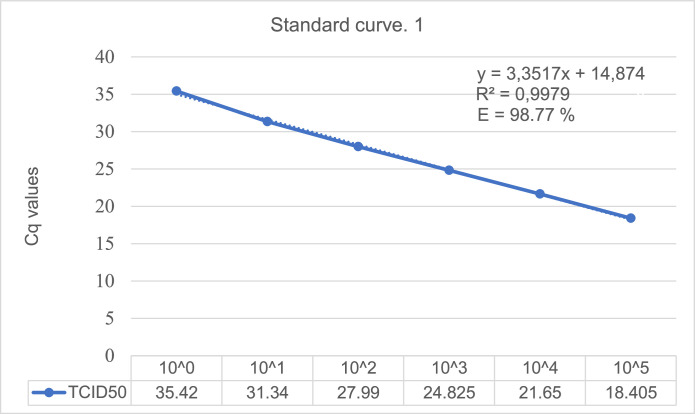
Standard curve 1 generated from RT-qPCR assays of the dilution series of extracted RNA from EV-D68. The ten-fold dilution was done with Rnase-free water before run in duplicates. The different TCID_50_ values and Cq values measured are presented for the six first dilutions. Dilution number six was the lowest concentration measured, excluding the other three.

**Fig. 4:**
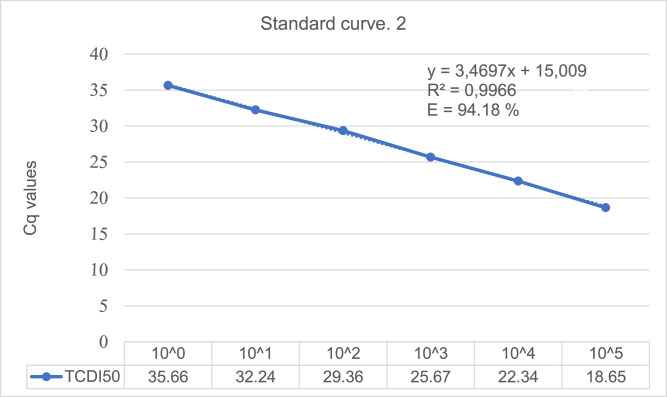
Standard curve 2 generated from RT-qPCR assays of the dilution series of extracted RNA from EV-D68. The ten-fold dilution was done with Rnase-free water before run in duplicates. The different TCID_50_ values and Cq values measured are presented for the six first dilutions. Dilution number six was the lowest concentration measured, excluding the other three.

The serial dilution creating standard curve 1 (Fig. 3) was linear with an R^2^ (correlation coefficient) value of 0.9979. Standard curve 2 (Fig. 4) was also linear with an R^2^ value of 0.9966. These R values indicate a stable method with efficiency calculated to be (10 ^(1/3.3517)^−1) * 100 = 98.77% for standard curve 1 (Fig. 3) and (10 ^(1/3.4697)^ −1) * 100 = 94.18% for standard curve 2 (Fig. 4)

All deviations are within one standard error of the best minimum quadratic regression line. The results showed a change in Cq for every tenth dilution (10x) with a mean (ΔCq mean) of 3.4 for both standard curves which are nearly 100% transcription efficiency.

### Sensitivity

For each replicate within a single standard curve, the standard deviation and coefficient of variation (CV) were calculated. This is found in *Appendix B* where the average value for all the standards also is included. The lowest concentration registered in the standard curve is set as the detection limit. The limit of detection (LOD) describes the assay's relative sensitivity. The value for LOD was set to Cq 18.405 equal to TCID_50_ 10^5^ from standard curve 1 (Fig. 3). The values ​​showed little variation in the Cq value for the same concentration which refers to the analytical sensitivity. The more similar the same Cq values are for the same replicates the better the analytic sensitivity is for the method.

### Reproducibility and repeatability

To evaluate the precision of each replicate within a single standard curve, standard deviations, and coefficient of variation (CV) were utilized intra-assay variations between the two analyses and the inter-assay variations between the replicates for the same analysis time showed stable Cq values ​​that describe the reproducibility and repeatability of the assay. Deviation within Cq +/- 0.5 was accepted. This was the case for all points apart from the 10^−1^ standard in preparation-2 (standard curve 2), which had a standard deviation of 0.42 Ct. The coefficient of variation was 0.01 or less for all standards in the dilution series.

TCID_50_ is converted into equivalent number of viral strands by estimating the Cq for a given TCID_50_ and then use the standard curve to determine specific amounts of virus based on the Cq values. Such analysis showed that Cq 18.5 corresponded to 10^−5^ TCID_50_ for EV-D68. The 10^−5^ dilution is equivalent to the virus TCID_50_ with a Cq value of 35.42 for standard curve 1 (Fig. 3) and 35.66 for standard curve 2 (Fig. 4). The most common TCID50 values normally lie in an application range between 2 TCID_50_ corresponding to 10^−3^ dilution and 3 TCID_50_ corresponding to 10^−2^ dilution.

### Specificity

The specificity of the method has been validated based on the experimental framework. The reagent has been investigated with samples from both infected and uninfected cells. No quantification was obtained for the uninfected samples. All practical work with EV-D68 has been done according to recommendations when working with the virus and carried out under sterile conditions [7]. It is therefore assumed with great certainty together with the results from the in-silico analysis, that the probe would solitarily detect EV-D68.

## Discussion

Here, we describe a quantitative real-time PCR as a distinct and widely used method for the diagnosis of virus infection compared to traditional cell culture and distinguishing cytopathic effect (CPE) [4,21]. Developing a good reverse transcription qPCR (RT-qPCR) will help with the detection and quantification of EV-D68 (HRV87). A good method makes it possible to separate EV-D68 infection from other virus infections. Because of the extensive genetic variability of EVs [4,^22^,23], there are limited RT-qPCR assays available for the detection of EVs, especially assays for detecting EV-D68 (HRV87) [24]. The performance evaluation of each assay was conducted in accordance with the MIQE guidelines.

The EV-D68 was propagated after the transfection of A549 cells with EV-D68. At the startup of virus cultivation, EV-D68 was inoculated at two different temperatures (33 °C and 37 °C) on the A549 cell to determine the optimal temperature for/the impact of temperature on the virus propagation. The temperatures considered were based on the virus's optimum growth conditions at 33 °C and the cell's optimal growth conditions at 37 °C and 5% CO_2_. The assessment for the virus propagation conditions was based on the development of infection and morphology of the cells. The cells displayed approximately the same growth rate and morphological characteristics at 33 °C as those at 37 °C. Meanwhile, the virus did not show stable development at 37 °C and 5% CO_2_. Based on these observations, all propagation of EV-D68 was performed at 33 °C.

In conclusion, a sensitive RT-qPCR assay for the detection of human enterovirus D68 is established and validated through detection in cell cultures. This study includes a detailed experimental setup for the direct method that is easy to implement. The described RNA extraction method is necessary and easy to use for isolating the viral RNA before the experimental setup on PCR plates. The instrumental setup in this study is also easy and straightforward. This RT-qPCR assay provides advantages with its rapid and closed tube system for EV-D68 identification. The developed assay in this study is restricted to detecting EV-D68 from standard samples and therefore is essential for research use. For the application of this method in clinical samples, the sensitivity and specificity need to be further assessed in future clinical studies.

## CRediT authorship contribution statement

**Hanne Lillerovde Ørstenvik:** Methodology, Investigation, Validation, Formal analysis, Writing – original draft. **Ann-Kristin Tveten:** Methodology, Software, Validation, Writing – review & editing. **Yanran Cao:** Writing – review & editing, Supervision.

## Declaration of Competing Interest

The authors declare that they have no known competing financial interests or personal relationships that could have appeared to influence the work reported in this paper.

## Data Availability

Data will be made available on request.

## Appendix Supplementary materials

Image, application 1

Image, application 2
